# The Etiology of Pyorrhœa Alveolaris Discovered so Stated

**Published:** 1900-07

**Authors:** B. F. Arrington

**Affiliations:** Goldsboro, N. C.


### THE ETIOLOGY OF PYORRHŒA ALVEOLARIS DISCOVERED SO STATED.

BY DR. B. F. ARRINGTON, GOLDSBORO, N. C.

There is recorded in the International Dental Journal for July, 1899, a paper written by Dr. W. J. Younger, and read by him before The New York Institute of Stomatology, April 4, 1899, in which he represents to have discovered the true cause of pyorrhœa, and reported cases (very extreme) treated and cured.

Dr. Younger is a prominent member of the dental profession, and doubtless is competent for successful execution of work in general practice in which superior skill is requisite, and to make bacterial investigations to some extent. But in this instance he has erred and blundered badly (an occasional occurrence with the most skilful and scientific), and must of necessity do his work of investigation over, and make it more thorough, if he wishes to establish his theory as truth beyond questioning.

The doctor expresses his convictions very pointedly as to what he believes to be the true etiology of pyorrhœa, but unfortunately cannot and does not find the cause until the disease has played havoc with the soft tissues and alveolus, and has almost run its course.

In reading the paper it will present as evident to the mind of any dentist at all familiar with the disease in question and interested in investigations for cause that Dr. Younger's findings are out of place and are misleading. Work of investigation for cause of a local disease must commence at location of first perception, the beginning, and not at the winding-up or terminus of a disease in a distant location. The locality of commencement of disease must first be determined; then take it in its incipiency, and follow its progress, and record carefully the varied features and changes for data and guidance.

In no disease is there better-defined features in the various stages of progress than in this disease, pyorrhœa alveolaris, so called.

I have long contended that the appellation *pyorrhœa alveolaris* was erroneous and misleading, and until discarded the disease would continue a mystery and a puzzle, and would not be diagnosed readily in the early stages and treated as successfully as it should be. It is unquestionably a pericemental trouble, commencing at the gingival margin, varying in features during progress to destruction of alveolus and loss of teeth. A disease must not be diagnosed and named by symptoms and features that are never present except in the advanced stages, as is the case generally with this disease. The deposits on the roots of teeth, pus formation, and waste of the alveolar process are never perceptible until the disease has made considerable progress. Hence the absurdity of the name, pyorrhœa alveolaris.

There must be, and is, a beginning, starting-point, with ever-present diagnostic features, in this, as in all defined local diseases. With this fact established, there is no reason why we should wander about in investigating for cause or question as to the point first to be investigated and treatment to be applied.

The name *gingiva pericementitis,* which better defines, locates, and makes more explicit the disease in its incipiency and early stages, would, I think, more correctly guide us. At the early stage of the disease, when it is just perceptible at the margin of the gum, it is definitely outlined with diagnostic marks, and is as distinctly a disease as when further advanced, and as many other diseases. The symptoms following in course of progress, deposits, pus formation, waste of membrane around the roots, and waste of alveolar process, are but sequences to be looked for if the disease is neglected.

In the advanced stage of every well-defined case of gingiva pericementitis deposits on roots of teeth and pyorrhœa will be recognized; they are never-failing features, and to effect a cure (treatment speedy or prolonged) without effectively removing the deposits is impossible. The removal must be accomplished with suitably shaped, *smooth* edge scalers. The application of acids to soften deposits is useless, as none so far has proved effective.

If treatment is commenced in the early stages, there will be neither deposits nor pus (pyorrhœa) to contend with, and cure can be easily effected.

The chief error committed by Dr. Younger in his work of investigation for cause of the disease consists in his beginning with the disease in the advanced stages, with the consequent symptoms, and not with the disease in its incipiency.

If a "specific bacillus" is the true cause of the disease, as stated by Dr. Younger, it certainly ought to be found and located at the gingival margin, the locality where the disease (not pyorrhœa) first makes its appearance.

Dr. Younger says, "What I personally consider pyorrhœa to be. It is characterized by an inflammation of the gums, a deposit of characteristic greenish-gray or slate-colored tartar, and wasting of the alveoli, accompanied by the formation of pus and pus pockets between the tooth and alveolus; the disease being due, as I believe, to a specific bacillus;" and says, "The cause of the deposits around the teeth is due to the presence of bacteria;" also, that "in the pus taken from the deep parts, the location where the active destruction of the alveoli is going on, numerous bacteria are found." Then he says, "Enough has been learned to satisfy us in this, at least: That we have a distinct, specific bacillus found in these true cases of pyorrhœa that is found only in those diseased portions which are not contaminated by septic material; in other words, it is, in my opinion, one of the normal bacteria of the mouth, rendered virulent through some agency which is not yet determined." He further says, "This bacillus is not found along the alveolar edge of the tooth; it is not found in the collections of tartar along the gingival margin; it is found only in the deep-seated pockets where the advance guard of the disease is progressing."

If this bacillus, of which Dr. Younger has written, is truly what he says he believes it to be,—one of the normal bacteria of the mouth,—how can it be a specific bacillus, and the cause of the disease? If the cause in question, it ought to be found along the alveolar edge and in the deposits along the gingival margins, and not only in the deep-seated pockets, for at the margins the disease commences, and the deposits along the gingival margins are the same in quality as those located lower down or farther up towards the apex of the roots.

If the doctor will renew and continue his investigations for more definite and convincing results, I think it more than probable he will view the subject differently, and will proclaim conviction that the "specific bacillus" of which he has written and represents to be the true cause of disease is nothing more nor less than normal bacteria much improved, developed, and vivified by migration and a plentiful supply of good nourishment, favorable to growth and action of bacilli, a consequence and not a cause. Such would be a reasonable conclusion, at least, until there can be produced some evidence of their presence at the gingival margin.

Ordinarily it is wisest and best not to herald a new theory, or discovery involving minute scientific work, until facts are established beyond a question of doubt; then no one would be led astray, and there would be less room or cause for questioning and controversy, and facts revealed through scientific investigation would be more universally acceptable, and progress would develop more rapidly.

Scientific investigations are daily developing to our minds new lights, beauties, and wonders. Developments most surprising and startling may, must be anticipated, and as science reveals and *establishes* new truths, we must accept and profit by them. But we must be cautious, and not hastily jump at or accept conclusions, nor take for granted that every new theory advanced and proclaimed to be evolved through *scientific* investigation is truth beyond questioning, for we have, to the contrary, too many evidences of error. Whenever there is evidence of inconsistencies and lack of thoroughness in the handling and presentation of any subject, there is just cause for questioning, and the sooner it begins the better for science and all concerned.

A knowledge of the true cause of gingiva pericementitis—pyorrhœa, if preferred—is desirable, and may possibly be revealed to us in course of time; so far it has not been. Yet we can diagnose the disease correctly, and treat successfully, by making it a point to begin at the beginning.

In reading Dr. Younger's paper, any one would reasonably infer that he deals with the disease in the chronic stage altogether. He says, "The treatment of pyorrhœa involves, first, the removal of every particle of the deposit; secondly, the maintenance of the tooth in rigid position while the process of repair around the root is going on; in other words, until the complete proliferation, attachment, and thorough organization of tissue has been established; and, thirdly, the restoration of attachment between the soft tissues of the alveolus and the root." He cites a case treated and cured after all the teeth had lost more than two-thirds of their alveolar support, and teeth "leaned in all directions, simply being held by their apical attachment." Also another case in which "all the upper incisors projected more than half their length from their sockets, retained only by apical attachment." These certainly were very extreme cases, and cures effected under such circumstances would evidence a feature of skill greatly to be admired and commended, and we will not doubt but that Dr. Younger could prove equal to such a task, but in this instance he failed. The doctor, like most of us, sometimes miscalculates, and through zeal and desire to effect a cure pronounces a cure before cure is complete, as is plainly evidenced in the case recited, in which there was so much loss of the alveolar process. The doctor says, "I had cured this gentleman of pyorrhœa, but his lower teeth were still in the embrace of ligatures waiting for them to become rigid, when he suffered from an attack of asthma complicated with other organic disturbances," when one day the patient called and wished him to look and see what had happened. He looked, and to his surprise he found an ugly state of things; treatment had to be renewed and continued for some time. The facts as stated by the doctor are proof positive that he was over-sanguine, and supposed a cure effected when it was not a cure. Had the cure been complete, as he imagined, there could not have occurred such a state of things in so brief a period; evidently it was a case of dismissal of patient before cure was complete, not an infrequent occurrence in practice,—none of us are exempt.

A cure is a cure, and, when definitely established, a case of asthma with other complications cannot in any way bring about or cause return of the disease. I think the doctor will so decide if he will carefully consider the nature of the disease.

The expression "apical attachment" is very indefinite, but my estimate of it is that when a tooth is held in place by such delicate support, immediate extraction is safest and best practice, for the chances of restoration to firmness in sockets through any line of treatment are very limited. A loosened tooth may be indefinitely retained in the mouth, but it is never advisable.

As regards the case reported, of incisors "projecting more than half their length from their sockets," it was a strange freak, certainly not a frequent occurrence with the disease. During many years' practice in treatment of the disease I have witnessed no such feature. The question arises, What became of the vacuums created by the protrusion of the roots from their sockets? Here is great room for surmising and speculation, but to shorten the matter, and for enlightenment without effort to obtain by investigation, we will ask that the doctor explain, as he restored the teeth to their sockets and left them with surrounding tissues in a healthy condition. We will not question the correctness of the doctor's report, but it is permissible and reasonable to presume the teeth would not long remain as replaced; nature's laws and the nature of this disease would combine against such a result.

This is a disease of frequent occurrence, but not so frequent as Dr. Younger represents. He says, "At least ninety per cent. of the population suffer with some form of the disease." Therein the doctor commits a blunder (many do). There is but *one form* of the disease, but there are sundry features that present as the disease progresses. I speak pointedly and positively on this subject, for such are my convictions, established through practice and watchful observation, and I feel assured that investigation and time will confirm the verity of my assertions. The percentage of cases of this disease in proportion to population is large, but nine per cent. in place of ninety would be a liberal estimate. All classes and grades of society are subject to it, and it seems to be on the increase, and is more common in remote rural sections and with the poorer and neglected classes in towns and cities than with the higher and better-to-do grades of society,—the cause evident upon reflection.

It is not dependent upon any particular state of the system for origin, for all alike, the robust and the feeble, seem to be equally subject to it; nor is it a consequence or in any way complicated with any other disease. It is independent, distinct, and well defined, always first perceptible at the margin of the gums, develops more and more plainly, with diagnostic features, and progresses (let the general state of the system be what it may) stage by stage, with features never varying, developing until it abates with destruction of the alveolar process and loss of teeth.

It is amenable to treatment, and a large percentage of cases can be cured if treatment is not too long delayed. In the early stages of the disease the treatment is simple, and need not be of long duration. In cases well advanced, deposits on roots and teeth loose, the first thing requisite is to determine the extent of waste of the alveolar process and the strength of teeth in sockets. In a majority of cases, when teeth are much loose and have lost more than two-thirds of the alveolar support, immediate extraction I have found to be wisest and best practice; also the extraction of all diseased and non-useful roots. This procedure is justified and advised, based upon the fact that the restoration of alveolar process when destroyed by this disease never can be effected for the support of teeth; and for another fact, that loosened teeth and diseased roots had better be out of the mouth than in, as such are always hurtful to health to some extent.

I have thoroughly tested the merits of lactic acid in the treatment of this disease, but have not been able to obtain such results as reported by Dr. Younger.

My experience in the use of sulphuric acid justifies me in saying that a very large percentage of cases can be cured within two weeks, a small percentage will require longer time, say from three to four weeks, a smaller percentage still cannot be cured by treatment, and the only remedy for relief is extraction.

I do not longer attempt to restore alveolar process, nor do I try to effect union of alveolar tissues with roots of teeth denuded of natural membrane by this disease. My efforts in that direction have proved a complete failure. I am thoroughly convinced that the most that can be accomplished by any process of treatment is to check the disease and establish a healthy state of osseous and soft tissues, and to so tone up and strengthen the gums as to effect a close fit to the roots of teeth, and by persistent, regular use of tooth-brush and moderate finger-pressure on gums as needed, and occasional application of a suitable tonic and astringent mouth-wash, to effect continued close proximity of soft tissues to roots to prevent ingress of particles of food during process of mastication. This, in my judgment, is the extent of possibilities of accomplishment by treatment in the advanced stages of the disease. It is needless and unwise for us to profess to be able to accomplish more in treatment of disease than is reasonable and possible. There is a limit to possibilities, and a line beyond which we cannot presume to venture with safety to the credit of the profession.

Further detail concerning this disease, as to treatment or otherwise, would make this paper too lengthy. But before closing I will mention for consideration several special features.

The disease never extends beyond definitely defined limits. The usual pain and discomfort attending abnormal conditions of the soft tissues of the mouth are but slightly conspicuous (sometimes not at all) with this disease.

Occlusion of the jaws with firmest pressure during process of mastication seldom causes a feeling of discomfort. A person is often troubled with the disease to the extent of deposits on roots and pus formation, and are not aware from any feeling of discomfort that the mouth is not in a healthy condition.

Pus follows deposits, and never ceases so long as deposits are adherent to roots. The deposits never attach except to the cementum. Enamel and smooth dentine are exempt. Ordinary tartar will, often does, adhere to the deposit peculiar to this disease, but the deposit of the disease never forms and adheres to ordinary tartar. The application of acids to soften the deposit is non-effective. No acid known will accomplish such a result in the mouth.

Some persons pass through life troubled with this disease for thirty or forty years, sustaining loss of many teeth, with freedom from any type of indigestion, and all the while enjoying comparatively good health. Others suffer in health until this disease is treated and cured, after which, improvement of general health is perceptible.

The disease seldom, if ever, commences after the age of forty. I feel confident I have never seen a case in its incipiency after that age.

When all other remedies fail to cure, much may be accomplished by judicious use of sulphuric acid.

